# NMR Assessment of the High Order Structure of Biological Therapeutics in Erythrocytes Provides a Tool for Drug Delivery Design

**DOI:** 10.1021/jacs.5c05617

**Published:** 2025-07-16

**Authors:** Luis Padilla-Cortés, Giulia Roxana Gheorghita, Francesco Currò, Rebecca Calamandrei, Bianca Susini, Sara Callozzo, Giulia Crivello, Pasquale Russomanno, Enrico Ravera, Linda Cerofolini, Marco Fragai

**Affiliations:** † Department of Chemistry Ugo Schiff (DICUS), 9300 University of Florence, Via della Lastruccia 3, 50019 Sesto Fiorentino, Italy; ‡ Centro di Risonanze Magnetiche (CERM), 9300 University of Florence, Via Luigi Sacconi 6, 50019 Sesto Fiorentino, Italy; § Consorzio Interuniversitario di Risonanze Magnetiche di Metalloproteine (CIRMMP), Via Luigi Sacconi 6, 50019 Sesto Fiorentino, Italy; ∥ Department of Agricultural and Food Sciences (DISTAL), 9296 University of Bologna, Piazza Goidanich 60, 475121 Cesena, Italy; ⊥ Giotto Biotech, s.r.l. Via Francesco Crispi 6, 50129 Firenze, Italy

## Abstract

An effective delivery system is crucial for ensuring the therapeutic efficacy of a drug. This is especially true for biological drugs, which possess unique physicochemical properties and complex pharmacokinetic profiles, and thus require a dedicated design. Whole erythrocytes, and more recently, nanoparticles derived from red blood cells (RBCs), have been used in preclinical studies to deliver biological therapeutics: their biocompatibility and extended circulation time help prevent immunogenicity and reduce clearance and toxicity. However, characterizing such complex systems poses challenges that complicate their development and optimization. We argue that NMR spectroscopy enables the monitoring of the preservation of the high order structure of the encapsulated proteins, as well as their concentration, thereby assisting in formulation design, development, and manufacturing.

## Graphical abstract

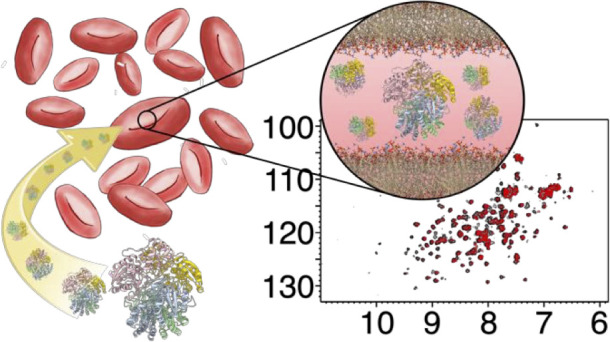

## Introduction

Several enzymes are currently in clinical use to treat a number of diseases. Relevant examples include enzymatic replacement therapy for genetically deficient patients, or the clearance of essential precursors for cancer cells.^1,2^ The effectiveness of these treatments is often limited due to the poor in vivo enzyme stability, immunogenicity, and the activity of inactivating antibodies. These challenges can be partially addressed by coating therapeutic proteins with biocompatible polymers such as PEG, which extend their half-life by increasing the size and shielding the enzyme from proteases and from the reticuloendothelial system.^3,4^ However, even the widespread used PEGylation can cause adverse reactions: hypersensitivity, immune reactions up to anaphylaxis, and cytoplasmic vacuolation have been documented for PEGylated protein delivery.^5,6^ The idea of using erythrocytes as enzyme carriers is even older than PEGylation and was first reported for beta-glucosidase and beta-galactosidase in the treatment of Gaucher’s disease.^7^ Erythrocytes are a promising delivery system because of their biocompatibility, biodegradability, prolonged circulation time, and ability to reduce side effects.^8,9^ The reason that brought to attention the possibility of using red blood cells is their intrinsic biocompatibility, because the patient’s own red blood cells can be utilized for encapsulation. Red blood cell membrane shelters therapeutic enzymes from the immune system action and plasma proteases, enhancing their therapeutic effects and enabling the safe administration of higher doses in the bloodstream.^10^ Over the years, the method of encapsulation using hypotonic dialysis has been automated to enhance both the efficiency and speed of loading proteins within red blood cells directly obtained from patients on-site.^11^ Additionally, various other loading techniques have been successfully implemented, such as utilizing low molecular weight protamine to facilitate the translocation of enzymes across the red blood cell membrane without causing significant disruption.^12,13^ More recently, RBC membrane-derived nanoparticles have been used as new drug delivery system.^14^ Several erythrocyte-encapsulated enzymes are currently under development, and one has undergone clinical evaluation.^15–17^

l-Asparaginase II was one of the first approved biologics used to treat acute lymphoblastic leukemia (ALL) and lymphoblastic lymphoma.^18,19^ This enzyme, derived from various sources, is still used in patients in its unmodified and PEGylated forms, and its encapsulated erythrocyte formulation (GRASPA) has been evaluated in clinical trials against ALL and pancreatic cancer.^20–22^ Although GRASPA’s approval for clinical use against ALL was discontinued, this therapeutic candidate remains a model for developing analytical tools to characterize this innovative class of biologics. To date, the characterization of erythrocyte-encapsulated enzymes has largely relied on assessing their enzymatic activity, either within the encapsulated formulation itself or after extracting the enzyme following cell lysis.^23^

The high order structure (HOS) of enzymes is a critical quality attribute because it defines the active conformation, allowing it to recognize and bind to its physiological substrates. This structural specificity is fundamental to the enzyme’s function, as it determines the catalytic efficiency. Therefore, assessing the preservation of the high order structure of the enzyme is important for ensuring its biological activity and therapeutic efficacy, as well as for a comprehensive understanding of its formulations and manufacturing design and process. Moreover, the folding state of a protein, or its degraded forms can influence their immunogenicity, thus posing a risk to the safety of protein therapeutics.^24^ Numerous studies have been published on the structural characterization of drug delivery systems; however, relatively few are focused on the assessment of the structural integrity of biologics inside these formulations.^25^ Given the structural complexity of biologics, NMR is a powerful tool for assessing the HOS of molecules in complex matrices or pharmaceutical formulations due to its atomic resolution and versatility.^26–31^ Solution NMR, with its short acquisition times and minimal sample preparation, is emerging as a crucial component in analytical workflows for biological therapeutics.^32–35^ Additionally, it enables the study of entire complexes in environments that closely mimic physiological conditions, facilitating the structural characterization of internalized cargo within the intact system and correlating it with biological functionality. Moreover, NMR has been widely used to investigate the structural features of proteins, peptides and nucleic acids embedded in confined environments, such as cells.^36–40^ This study demonstrates how the NMR characterization of three erythrocyte-encapsulated proteins offers insights into their HOS preservation and a semiquantitative assessment of the encapsulation yield. This represents a significant contribution to potentially optimizing encapsulation protocols, sample handling, and storage in erythrocytes and RBC membrane-derived nanoparticles.^41^

## Results

### Analysis of the High Order Structure of Proteins Encapsulated within Erythrocytes

Three proteins differing in pharmacological relevance, function, size, and structural features were selected to investigate their encapsulation within RBC. ^15^N-enriched human carbonic anhydrase II (CAII),^42^ ^15^N-enriched human transthyretin (TTR),^43^ and ^2^H, ^13^C, ^15^N-enriched l-Asparaginase II (ANSII)^4,44^ were encapsulated in bovine red blood cells and used as models for the characterization of the preservation of the HOS of the proteins in such a delivery system. Protein encapsulation was achieved using the hypotonic dialysis method.^45^ Briefly, bovine erythrocytes, mixed with concentrated protein solutions, were first dialyzed against a hypotonic solution to promote membrane permeability and protein encapsulation, then against isotonic solutions with membranes of different pore sizes to reseal the red cells and remove the nonencapsulated protein (see the details in the Materials and Methods section). During this process, we observed that a portion of the hemoglobin and likely some cytoplasmic components were released into the external buffered solution. Encapsulation and structural characterization were monitored using 2D ^1^H–^15^N SOFAST-HMQC NMR experiments that are optimized for increased sensitivity by using band-selective pulses for proton amide excitation, leading to shorter longitudinal relaxation and reduced recycling delay.^46^

CAII was used to assess the effectiveness of the selected methodology in encapsulating proteins within RBC. ^15^N-filtered spectra were recorded (Figure 1) on samples prepared using three different protocols: (i) the dialysis protocol for encapsulation in the presence of the ^15^N-labeled CAII (Encapsulated CAII); (ii) the dialysis protocol for encapsulation in the absence of the ^15^N-labeled CAII (Control 1); (iii) ^15^N-enriched CAII added to RBC without performing the hypotonic dialysis to load the protein (Control 2). The intensity of the NMR signals from the first FID of the 2D ^1^H–^15^N SOFAST-HMQC spectra of Control 1 and 2 were compared with that of a sample of ^15^N-enriched CAII encapsulated in RBC following the correct protocol described in Materials and Methods section. The spectrum recorded on the sample (Encapsulated CAII) prepared following the protocol showed intense signals in the amide proton region, while only weak background signals of HN protons were visible in the spectra of the two controls (Control 1 and Control 2). These results suggest that the background signals of HN protons, resonating in the range of 7 to 9 ppm in the spectra of the controls, originate from hemoglobin present in RBCs. Indeed, despite the low natural abundance of ^15^N (∼0.4%), the physiological concentration of hemoglobin in erythrocytes is high (∼3 mmol dm^–3^), therefore its signals could be detected.^47^ Additionally, the supernatant of the last rinse step was always analyzed through NMR to check for the presence of residual free protein. The absence of signals in the spectra confirms that the cross-peaks observed in the 2D spectra of the samples are related to the encapsulated proteins only (Figures S1 and S2).

**Figure 1:**
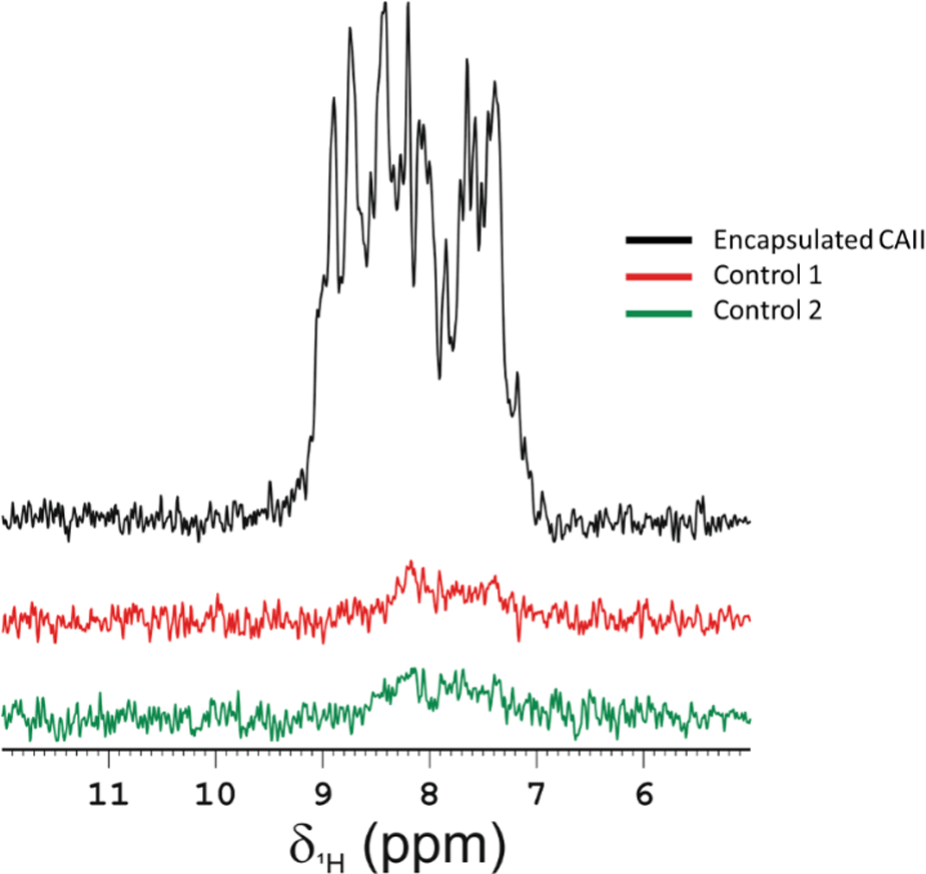
Evaluation of the efficacy of the encapsulation process for ^15^N CAII. 1st FID of the 2D ^1^H–^15^N SOFAST-HMQC of encapsulated ^15^N CAII in RBC (black), RBC undergoing the dialysis protocol for encapsulation in the absence of the ^15^N-labeled CAII (Control 1, red) and RBC undergoing the encapsulation process with ^15^N CAII without performing the hypotonic dialysis to load the protein (Control 2, green). The spectra show successful encapsulation of ^15^N CAII in RBC using the protocol and absence of the protein in the controls. The spectra were acquired at 310 K on a spectrometer operating at 1200 MHz (^1^H Larmor frequency).

The 2D ^1^H–^15^N SOFAST-HMQC spectra recorded on the three proteins encapsulated within the RBCs were of relatively good quality.^48^ The reduced amount of cytoplasmic components and hemoglobin, because partially released from RBCs during the hypotonic dialysis, likely accounts for the relatively good resolution of the spectra. The backbone resonances in the spectra were superimposable with those of “free” nonencapsulated proteins (Figure 2), demonstrating the preservation of the high order structure of all the proteins upon encapsulation in RBCs. Furthermore, assignment of the visible cross-peaks was easily obtained by comparing the assignment available for free CAII,^42^ ANSII,^4^ and TTR^43^ with the 2D ^1^H–^15^N SOFAST-HMQC spectra acquired on the encapsulated enzymes (Figure 3). Almost all the residues of CAII (98% over the available assignment) could be reassigned (Figure 3, panels A and B), further confirming the preservation of the high order structure of encapsulated CAII. The 76% of the residues over the available assignment could be reassigned for ANSII (Figure 3, panels C and D). In the case of TTR, the signals in the 2D ^1^H–^15^N SOFAST-HMQC are affected by a larger line broadening due to the protein size (*M*_w_ 55 kDa) and the absence of deuteration both for the free and encapsulated protein. However, the quality of the spectra of the protein inside the cells is comparable to that of free TTR and overall, although in severe overlap, most of the signals (90% over the available assignment) could be reassigned (Figure 3, panels E and F).

**Figure 2:**
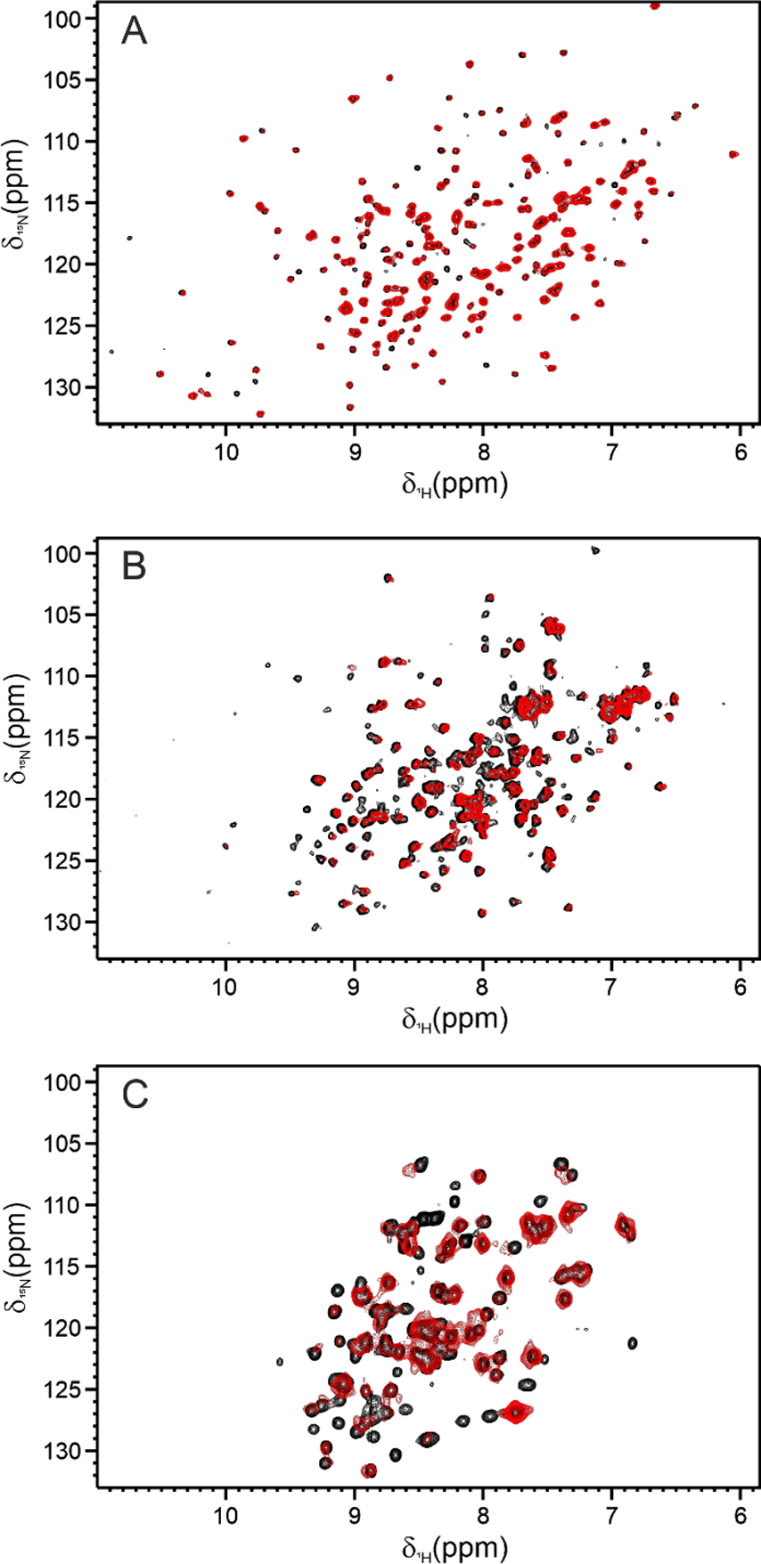
Comparison of the NMR spectra between free and RBC-encapsulated proteins. 2D ^1^H–^15^N SOFAST-HMQC NMR spectra of (A) CAII encapsulated in RBC (red) and free CAII (black), (B) ANSII encapsulated in RBC (red) and free ANSII (black), (C) TTR encapsulated in RBC (red) and free TTR (black). The spectra of CAII and ANSII were acquired on a spectrometer operating at 900 MHz, while the spectra of TTR were acquired on a spectrometer operating at 1200 MHz. All the experiments were recorded at 310 K in PBS buffer, pH 7.4.

**Figure 3:**
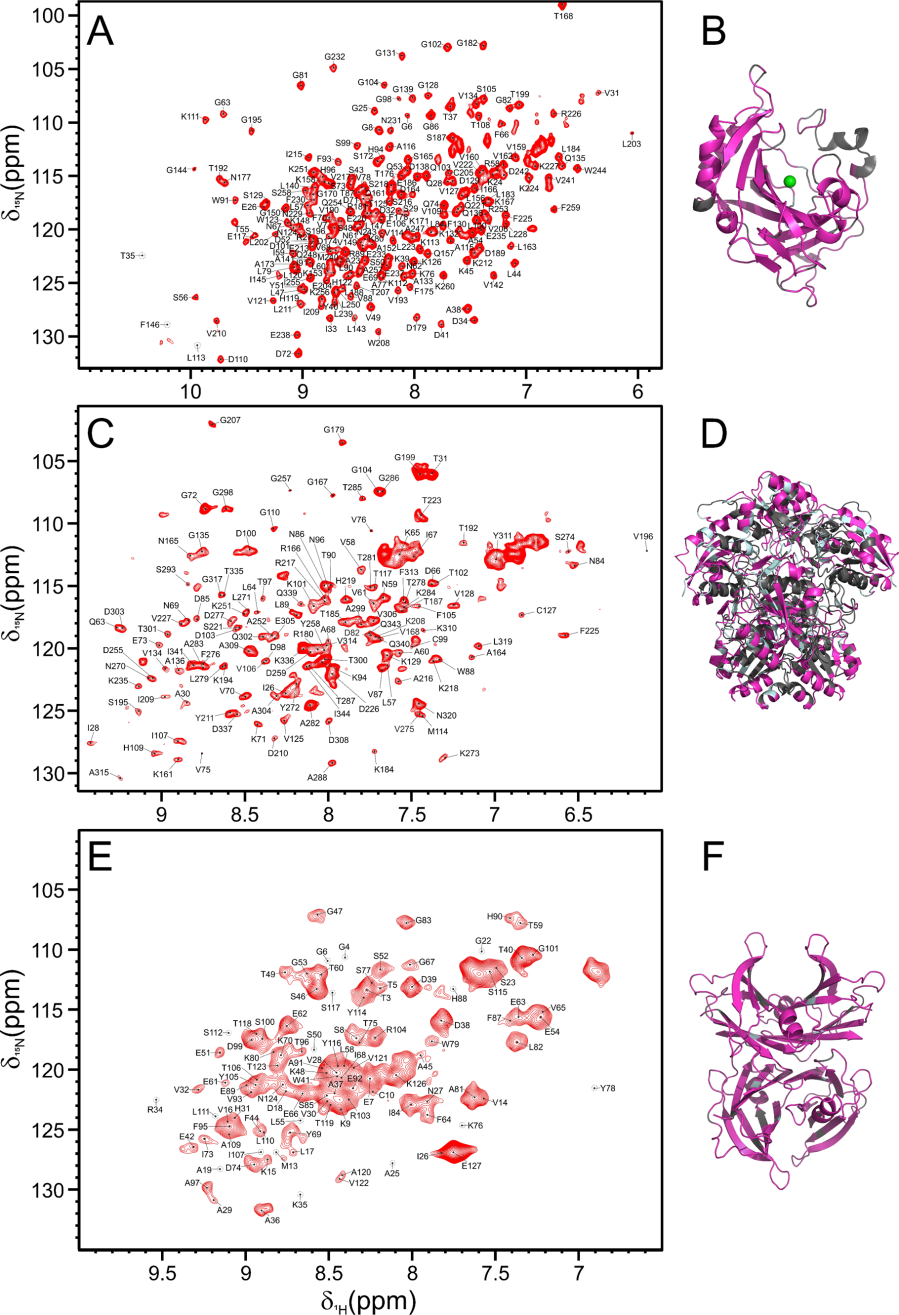
Assignment of RBC-encapsulated proteins and their X-ray structure highlighting the reassigned residues. (A–C–E) 2D ^1^H–^15^N SOFAST-HMQC spectrum of proteins encapsulated in red blood cells with the assignment indicated on the signals, respectively CAII (A), ANSII (C) and TTR (E). (B–D–F) X-ray structure of proteins in light blue, the residues for which the resonances have been reassigned for the protein encapsulated in the red blood cells are highlighted in magenta and the residues neither assigned in the free nor in encapsulated proteins are highlighted in gray. The PDB codes for obtaining the X-ray structures were (B) 3KS3 for CAII, (D), 3ECA for ANSII and (F) 1BMZ for TTR.

In general, the spectra of the proteins encapsulated inside the RBCs and acquired immediately after sample preparation are of relatively good quality; however, broader cross-peaks were observed due to the intrinsic line-broadening effect of proteins within cells.^49–52^ This could be caused by (a) a crowded and more viscous environment that causes molecules to diffuse slowly, leading to increased transverse relaxation rates (R_2_); (b) weak interactions with the cytoplasmic components; (c) magnetic field inhomogeneities related to the internal heterogeneous structure of the RBCs; and primarily (d) the presence of deoxyhemoglobin, and the oxidative stress in erythrocytes over time that induces the formation of methemoglobin and hemosiderin, where Fe^2+^ is oxidized to Fe^3+^ that has a more detrimental effect on spectral quality.^53–55^ This latter process contributes to a comprehensive degradation of the sample within the magnet at 310 K and to the broadening of signals over time. The broadening of CAII signals over time is shown in Figure S3. The increase of line broadening for the encapsulated protein was assessed on CAII. ^15^N transverse relaxation rates were estimated on free CAII in PBS and CAII encapsulated in RBCs by recording the first FID of Carr–Purcell–Meiboom–Gill (CPMG) experiments. An average transverse relaxation rate (R_2_) of 15 ± 1 and 22 ± 6 s^–1^ was found for free and encapsulated CAII, respectively (Figure S4). These data confirm faster decay of magnetization for the encapsulated protein.

To demonstrate the sensitivity of this method to minor structural changes in proteins, we conducted an NMR analysis. This involved mixing an aliquot of free carbonic anhydrase II (CAII) with an aliquot of CAII that had been inhibited with furosemide, both at similar protein concentrations, prior to encapsulation in red blood cells (RBCs). The free and inhibited species can be distinguished by their different chemical shifts also when the protein is encapsulated in RBCs (Figure 4). The relative amount between the two species is almost conserved during the encapsulation process (55% of free CAII: 45% of inhibited CAII).

**Figure 4:**
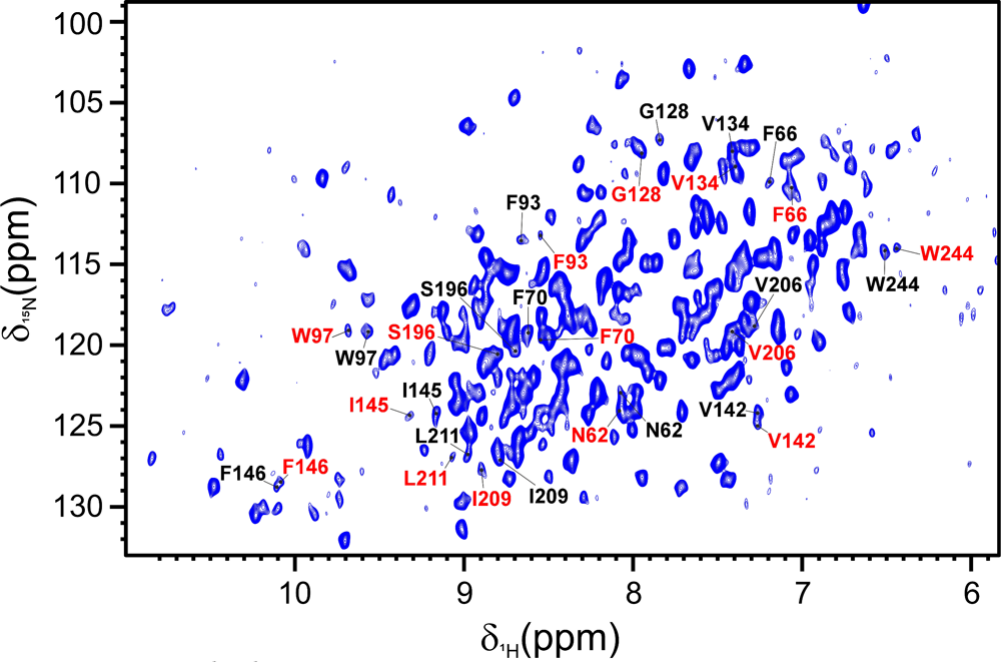
A 2D ^1^H ^15^N SOFAST HMQC spectrum was obtained for carbonic anhydrase II (CAII) encapsulated in red blood cells (RBCs), both in its free form and when inhibited by furosemide. The figure presents the assignment of shifted signals, with signals from the free protein indicated in black and those from the inhibited protein shown in red. These spectra were recorded at 310 K on a spectrometer operating at 900 MHz (^1^H Larmor frequency).

Additional experiments on CAII were carried out using an instrument operating at 600 MHz. Although the signal-to-noise ratio is lower than the spectra recorded at high field (900 MHz), the signals of the protein are still visible and comparable (Figure S5). It is important to note that we observed variations in the quality of the spectra obtained from one preparation to another for the same protein. These differences are likely due to several factors, including the varying concentrations of the protein, challenges in reproducibility during the preparation process, particularly concerning the number of red blood cells treated and introduced into the NMR tube, and possibly the concentration of paramagnetic species. Tuning these parameters is an essential step to ensure the reproducibility of RBC as a delivery system, and thus its applicability in the pharmaceutical industry.

### Quantification of Encapsulated Protein

An estimation of the encapsulation efficiency of enzymes within RBCs can be obtained by comparing the intensity of signals of encapsulated protein in RBCs with those of free one of known concentration in the same buffer. A solution 18 μmol dm^–3^ of free ANSII was compared with the ANSII-RBC complex. The same NMR acquisition parameters were used to compare the NMR signal from the first FID of the 2D ^1^H–^15^N SOFAST-HMQC for both spectra (Figure 5). Given the absence of severe line-broadening on the NMR signal from the encapsulated ANSII, we could assume that the different relaxation properties of ANSII within the RBC complex do not significantly affect the integral calculation. The ANSII-RBC signals were 1.4 times more intense than the free ANSII signals. Therefore, we could estimate the concentration of encapsulated ANSII to be approximately 25 μmol dm^–3^. Considering that the initial concentration of ANSII in the solution used for encapsulation in RBC was 236 μmol dm^–3^, we estimate an encapsulation efficiency close to 10%, which aligns with literature values.^56^

**Figure 5:**
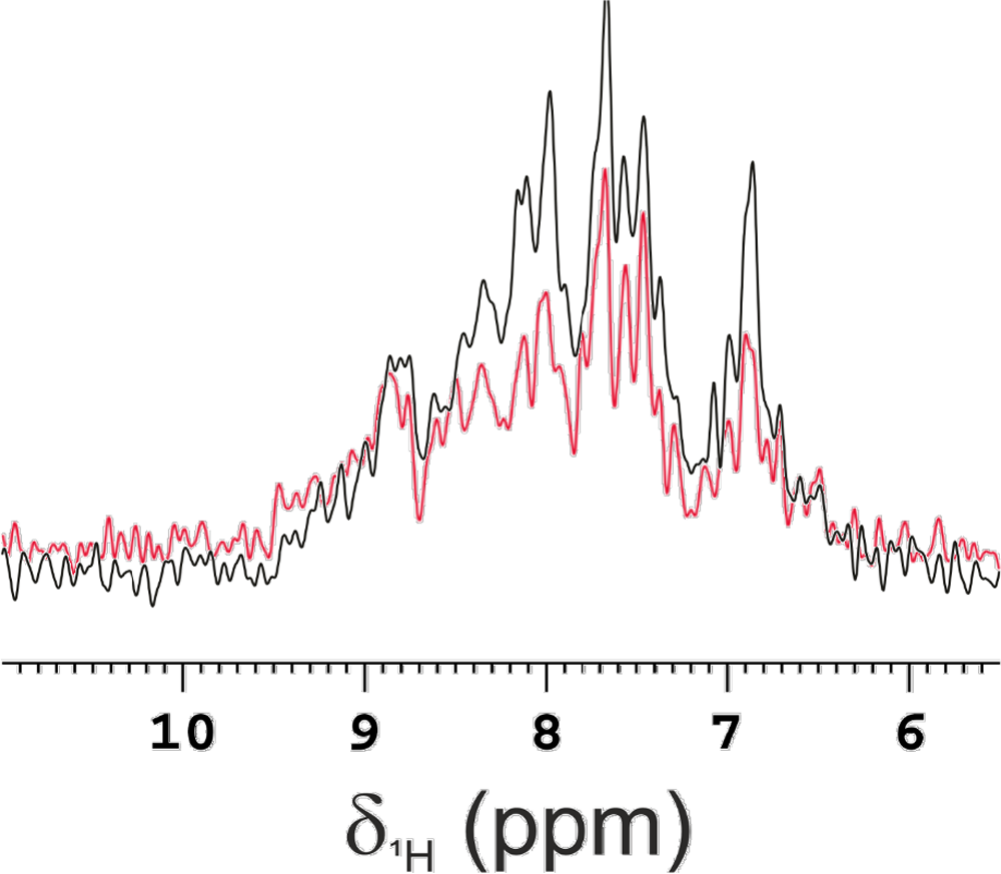
Estimation of ANSII content within RBC. NMR signal from the 1st FID of the ^1^H–^15^N SOFAST-HMQC of encapsulated ANSII (black), recorded immediately after sample preparation, and a solution of free ANSII 18 μmol dm^–3^ (red). The spectra were acquired at 310 K on a spectrometer operating at 1200 MHz (^1^H Larmor frequency).

## Discussion

The analysis of NMR spectra of protein therapeutics is a widely recognized method for evaluating the preservation of their structural integrity. Biological assays can provide the amount of biologically active protein. For instance, enzymatic assays are employed to assess the quantity of encapsulated active enzyme. However, these assays do not provide information on the presence of inactive enzyme, which may result from partial unfolding, alterations in the active site, or degradation. This information is particularly relevant because the folding or degradation state of a protein significantly influences its immunogenicity.

In-cell NMR studies have demonstrated that the quality of the NMR spectra is significantly influenced by the characteristics of the proteins expressed or internalized within the cells. Considering the evident similarities between the proteins incorporated into RBCs and those examined in in-cell NMR investigations, this study identified three proteins of pharmacological significance to validate the applicability of the proposed methodology. CAII, also physiologically present in erythrocytes, served as an optimal model for the initial optimization of the encapsulation protocol due to its stability and high expression levels. Its high molecular weight and globular structure place it at the boundary of systems that can be studied without deuteration. As a metalloenzyme, it also enables us to evaluate the possible effects of the cellular environment on the protein-metal stability at the high concentrations achieved in this delivery system. The spectra recorded on CAII showed that the protein maintained its native conformation upon encapsulation, as evidenced by the superposition of the resonances in the 2D ^1^H–^15^N SOFAST-HMQC NMR spectra of the free and encapsulated proteins. Almost all the resonances on the 2D ^1^H–^15^N SOFAST-HMQC spectrum of the encapsulated protein corresponding both to secondary structure elements and loops were easily reassigned, further proving the preservation of the native HOS. CAII has also been used to show the capability of the proposed method to detect the minor structural alterations due to the binding of an inhibitor to the active site of enzymes. The results presented here demonstrate the effectiveness of the NMR method in identifying and quantifying the fraction of inactive enzyme present alongside the active enzyme. Notably, the analysis of the mixture reveals that the chemical shift variations caused by encapsulation are minimal compared to those observed when a ligand interacts with the active site of the enzyme, or when there is unfolding or degradation.

Transthyretin has been considered in this study for its potential applications as drug carrier and additional drug delivery system.^43^ TTR, a protein with a complex quaternary structure, is composed of four identical subunits arranged as a dimer of dimers, resulting in a total mass of 55 kDa. Maintaining the tetrameric structure is crucial for its function as a carrier because it features a long channel that spans the entire molecule and contains two binding sites that can accommodate drugs and prodrugs.^44^ Despite the size of the protein, which causes line broadening of the signals and somewhat compromises the quality of the spectra also, in buffer solution, the superimposition of the 2D ^1^H–^15^N SOFAST-HMQC spectra shows that the protein retains its structural integrity upon encapsulation without dissociation into the constitutive monomers. More relevant is the study carried out on l-Asparaginase II, whose RBC formulation stands as a valuable model for the analytical characterization of erythrocyte-encapsulated enzymatic therapies.^12,21^ An in-depth investigation into the effects of l-Asparaginase II encapsulation on red blood cells has already been conducted: processed red blood cells (RBCs) showed dehydration, minor morphological changes, and metabolomic shifts but maintained a similar proteomic profile and improved osmotic resistance despite reduced deformability. The encapsulation process did not significantly affect RBCs half-life in a mouse model, suggesting their applicability as drug carriers for extended circulation time of drugs like asparaginase.^45^ However, encapsulation effects on the structure of the enzyme have yet to be investigated. Therefore, our solution NMR analysis enabled us to complete the characterization of the complex, confirming the preservation of the high order structure of l-Asparaginase II after encapsulation. The superimposition of the NMR spectra of free enzyme and erythrocyte-encapsulated ANSII confirmed the protein structural integrity within RBCs. Moreover, the quantitative analysis of ANSII encapsulated within red blood cells highlights the efficacy of using NMR spectroscopy to estimate encapsulation efficiency. By comparing the NMR signals from encapsulated ANSII with a reference solution of known concentration, we successfully estimated the degree of encapsulation, finding an efficiency close to 10% which aligns well with existing literature. The ability to perform this analysis on the pharmaceutical formulation with minimal sample preparation and relatively short acquisition times represents a further advantage for the development and optimization of pharmaceutical formulations.

## Conclusions

The development of an easily accessible methodology for the structural characterization of proteins encapsulated inside erythrocytes and red cell-derived nanoparticles represents a critical step for advancing and refining red blood cell-based drug delivery systems. Although the requirement for isotopically enriched proteins makes this methodology unsuitable for real-time monitoring of RBCs autologous preparations, this study demonstrated that NMR allows for a quick and straightforward assessment of the HOS preservation of erythrocyte-encapsulated proteins with minimal sample manipulation. Although the quality of the spectra does not allow for a detailed analysis of all the changes typically examined in comparability studies carried out in solution, this analysis still reveals any significant alterations that impact the structure of the encapsulated proteins and, consequently, their function. The analysis carried out on two enzymes and a carrier protein, different for molecular weight, structure, and function, shows that all the proteins retain their native structural features. The same NMR data can be used for fast, nondestructive quantification of the encapsulated protein unrelated to enzymatic activity and for general application. Consequently, despite the limitations still existing for labeling and size of many therapeutic proteins, this methodology is an important tool for designing, optimizing, formulating, and manufacturing novel red blood cell-based drug delivery systems.

## Materials and Methods

### Materials

Luria–Bertani (LB) medium, ampicillin, kanamycin, d-glucose, sodium chloride (NaCl), sodium phosphate dibasic (Na_2_HPO_4_), potassium phosphate monobasic (KH_2_PO_4_), magnesium sulfate (MgSO_4_), calcium chloride (CaCl_2_), ^15^N-amonium sulfate (NH_4_)_2_SO_4_, zinc sulfate (ZnSO_4_), Tris sulfate (Tris-SO_4_), isopropil-β-D-1-tiogalattopiranoside (IPTG), sodium phosphate dibasic heptahydrate (Na_2_HPO_4_·7H_2_O), sodium phosphate monobasic monohydrate (NaH_2_PO_4_·H_2_O), Tris hydrochloride (Tris-HCl), dithiothreitol (DTT), hydrochloric acid (HCl), sodium hydroxide (NaOH), ^2^H–^13^C-^15^N-enriched medium, Coomassie blue, ethylenediaminetetraacetic acid (EDTA), sucrose, reduced glutathione and ATP were purchased from Sigma-Aldrich. Plasmids were obtained from Twist Bioscience, San Francisco, CA.

### Expression and Purification of Uniformly Isotopically Enriched Carbonic Anhydrase II [U–^15^N]

The gene encoding α-CAII into the pCAM vector was transformed into BL21­(DE3) cells, which were subsequently precultured overnight in LB medium containing ampicillin (0.1 mg cm^–3^) and 1% glucose at 37 °C and 160 rpm. The main culture (1 dm^3^) was then incubated at 37 °C, 160 rpm until it reached the optical density at 600 nm (OD_600_) ∼0.6 and harvested for 15 min at 4000 rpm to proceed with the isotopic enrichment according to the Marley method. The cell pellet was resuspended in 1 dm^3^ of M9 minimal medium (3 g dm^–3^ KH_2_PO_4_, 0.5 g dm^–3^ NaCl, 6.8 g dm^–3^ Na_2_HPO_4_) supplemented with 2 mmol dm^–3^ MgSO_4_, 0.2 mmol dm^–3^ CaCl_2_, 3 g dm^–3^ glucose, 1.2 g dm^–3 15^N-ammonium sulfate, 0.5 mmol dm^–3^ ZnSO_4_ and ampicillin. The new culture was further incubated for 30 min at 37 °C and 160 rpm, before inducing with 1 mmol dm^–3^ IPTG and incubating overnight at 25 °C and 160 rpm. The cell culture was harvested at 7500 rpm for 20 min (JA-10 Beckman Coulter). The cell pellet was resuspended in 70 cm^3^ of 20 mmol dm^–3^ Tris-SO_4_, 500 μmol·dm^–3^ ZnSO_4_ pH 8 and underwent 10 cycles of sonication for 30 s with a resting period of 3 min on ice, 60% power (sonicator Vibra-cell of Sonics & Materials Inc.). The lysate was ultracentrifuged at 40,000 rpm for 40 min (Beckman Optima LE-80K Ultracentrifuge, rotor F15–6 × 100y from Thermo Scientific) and the supernatant recovered is filtered with a 0.45 μm filter. The supernatant, containing crude α-CAII protein, was purified by Nickel affinity chromatography using a linear 0–0.5 mol dm^–3^ Imidazole gradient on a HisTrap 5 cm^3^ (GE Healthcare). Finally, the α-CAII was subjected to a size-exclusion chromatography on a Superdex 75pg 26/60 column (Amersham Biosciences) in 50 mmol dm^–3^ sodium phosphate buffer at pH 7 (7.744 g dm^–3^ of Na_2_HPO_4_·7H_2_O, 2.913 g dm^–3^ of NaH_2_PO_4_·H_2_O, pH adjusted with HCl and NaOH).

### Expression and Purification of Uniformly Isotopically Enriched TTR [U–^15^N]

 BL21­(DE3) RIPL cells were transformed with pET-28a­(+) plasmid coding for TTR gene. The cells were cultured in ^15^N-enriched M9 minimal medium supplemented with kanamycin at 37 °C until OD_600_ reached 0.6–0.8 and then 1 mmol dm^–3^ IPTG was added for induction. The cells were incubated overnight at 37 °C and then harvested by centrifugation (JA-10 Beckman Coulter) at 7500 rpm for 15 min at 4 °C. The pellet was suspended in 20 mmol dm^–3^ Tris-HCl, pH 8.6 buffer supplied with 5 mmol dm^–3^ DTT (80 cm^3^ per liter of culture) and sonicated at 4 °C for 10 cycles of 30 s ON and 3 min OFF, at 60% power. The suspension was centrifuged at 40,000 rpm (Beckman Optima LE-80K Ultracentrifuge, rotor F15–6 × 100y Thermo Scientific) for 40 min and the pellet was discarded. The protein was purified by anionic-exchange chromatography using a HiPrep Q FF 16/10 column (GE Healthcare Life Science), previously equilibrated with 20 mmol dm^–3^ Tris-HCl, pH 8.6. The protein was eluted in 20 mmol dm^–3^ Tris-HCl, pH 8.6 with a linear 0–1 mol dm^–3^ NaCl gradient. Fractions containing pure TTR were joined and purified by Size Exclusion Chromatography using HiLoad Superdex 75pg 26/60 in 50 mmol dm^–3^ phosphate buffer, pH 7.5.

### Expression and Purification of Uniformly Isotopically Enriched l-Asparaginase II [U-^2^D-^13^C–^15^N]

 (DE3) C41 cells were transformed with pET-21a (+) plasmid with ANSII insert. The cells were cultured at 37 °C in ^2^H–^13^C-^15^N-enriched medium (-OD2 rich growth media, Silantes) supplemented with ampicillin until OD_600_ 0.6 was reached, then 1 mmol dm^–3^ IPTG was added for induction. All reagents were previously dissolved in ^2^H_2_O. The culture was incubated at 37 °C overnight and then harvested by centrifugation at 6500 rpm (JA-10 Beckman Coulter) for 15 min at 4 °C. The pellet was suspended in 10 mmol dm^–3^ Tris-HCl, pH 8.0, 15 mmol dm^–3^ EDTA, 20% sucrose buffer (60 cm^3^ per liter of culture) and incubated at 4 °C for 20 min under magnetic stirring. The suspension was centrifuged at 10,000 rpm (Beckman Optima LE-80K Ultracentrifuge, rotor F15–6 × 100y, Thermo Scientific) for 30 min and the supernatant was discarded. The recovered pellet was resuspended in H_2_O milli-Q (60 cm^3^ per liter of culture) and incubated at 4 °C for 20 min under magnetic stirring. Again, the suspension was centrifuged at 10,000 rpm for 30 min and the pellet was discarded. The supernatant was treated with ammonium sulfate to trigger the precipitation of ANSII. Under magnetic stirring, solid ammonium sulfate was added in aliquots up to 50% saturation. The precipitate was removed by centrifugation and discarded, then additional ammonium sulfate was added up to 90% saturation to trigger the precipitation of ANSII. The precipitated ANSII was redissolved in a minimal amount of 20 mmol dm^–3^ Tris-HCl, pH 8.6, and dialyzed extensively against the same buffer. ANSII was purified by anionic-exchange chromatography using a HiPrep Q FF 16/10 column (GE Healthcare Life Science). The protein was eluted in 20 mmol dm^–3^ Tris-HCl, pH 8.6 with a linear 0–1 mol dm^–3^ NaCl gradient. Fractions containing pure ANSII were identified by Coomassie staining SDS-PAGE gels, then joined and dialyzed extensively against the final buffer.

### Encapsulation of ^15^N-Labeled Proteins in Red Blood Cells

Commercially available Bovine red blood cells Packed 100% from Innovative Research were used. RBCs were washed by diluting 5 cm^3^ of 100% packed RBC in 5 cm^3^ of cold PBS. The sample was centrifuged at 2305 rpm (Heraeus Megafuge 16R Centrifuge, TX-400 Swinging Bucket Rotor, Thermo Scientific) for 1 min at 4 °C and the supernatant was removed. The process was repeated two more times.

^15^N-labeled CAII, TTR and ANSII were encapsulated using a previously reported hypotonic dialysis method of encapsulation.^57^ Seven parts (700 mm^3^) of washed packed RBC were mixed with 3 parts (300 mm^3^) of a concentrated solution of the protein (3 mmol dm^–3^ solution of CAII, 1 mmol dm^–3^ solution of TTR and 236 μmol dm^–3^ solution of ANSII). One cm^3^ of this suspension was placed on a dialysis tube (12 kDa MWCO) and dialyzed against 150 cm^3^ of hypotonic buffer (5 mmol dm^–3^ KH_2_PO_4_, 5 mmol dm^–3^ K_2_HPO_4_, pH 7.4) at 4 °C with a rotation of ∼20 rev/min for 180 min. The erythrocytes were then resealed by transferring the dialysis tube into a container holding 150 cm^3^ of isotonic buffer (PBS 1×, 5 mmol dm^–3^ glucose, 5 mmol dm^–3^ MgCl_2_) at 37 °C under continuous stirring for 60 min. The addition of reduced glutathione (3 mmol dm^–3^) and ATP (2 mmol dm^–3^) in the buffer was also evaluated (see Figure S6). An aliquot of CAII was also titrated with the active-site ligand furosemide. Then 1.95 cm^3^ of a solution of free CAII at the concentration of 330 μmol dm^–3^ was mixed with 1.89 cm^3^ of a solution of inhibited CAII at the concentration of 330 μmol dm^–3^. The volume of the resulting solution was reduced to 340 mm^3^. The same procedure for protein encapsulation was also performed on this sample.

In order to wash away nonencapsulated ^15^N isotopically enriched protein or released components from the erythrocytes, the sample was transferred into a dialysis tube of 1000 kDa MWCO and dialyzed against 1 dm^3^ of isotonic buffer at 4 °C for 12 h. This dialysis was repeated 2 times. The dialyzed erythrocytes were then washed with an equal volume of cold PBS three times. A first FID of 2D ^1^H ^15^N SOFAST-HMQC spectrum of a sample from the last wash was acquired in order to confirm the absence of any ^15^N-labeled protein outside of the red blood cells.

Two distinct control experiments were conducted using CAII, the first protein to be studied and used to verify the efficacy of the protocol. These experiments aimed to rule out the possibility of protein adsorption on the surface of the red blood cells and to confirm the efficacy of the washing procedure.

In Control 1, the erythrocytes were subjected to hypotonic dialysis in the absence of proteins. The samples were prepared by mixing 7 parts of washed packed RBC with 3 parts of cold PBS, then 1 cm^3^ of cell suspension was placed on a dialysis tube (12 kDa MWCO). The sample was dialyzed against 150 cm^3^ of hypotonic buffer at 4 °C with a rotation of around ∼20 rev/min for 180 min. The dialysis tube was then transferred to a container holding 150 cm^3^ of isotonic buffer at 37 °C under continuous stirring for 60 min. In order to wash away the released components from the erythrocytes, the sample was transferred into a dialysis tube of 1000 kDa MWCO and dialyzed against 1 dm^3^ of isotonic buffer at 4 °C for 12 h. This dialysis was repeated two times. The dialyzed erythrocytes were then washed with an equal volume of cold PBS three times.

In Control 2, proteins were added but not encapsulated. The samples were prepared by mixing 7 parts of washed packed RBC with 3 parts of a 3 mmol dm^–3^ solution of the ^15^N-labeled CAII, then 1 cm^3^ of cell suspension was placed on a dialysis tube (12 kDa MWCO). The sample was dialyzed against 150 cm^3^ of isotonic buffer at 4 °C with a rotation of around ∼20 rev/min for 180 min. The sample was then dialyzed against the same isotonic buffer at 37 °C under continuous stirring for 60 min. In order to wash away the nonencapsulated ^15^N isotopically enriched protein, the sample was transferred into a dialysis tube of 1000 kDa MWCO and dialyzed against 1 dm^3^ of isotonic buffer at 4 °C for 12 h. This dialysis was repeated two times. The dialyzed erythrocytes were then washed with an equal volume of cold PBS three times.

### Sample Preparation and NMR Measurements

All NMR experiments were recorded at 310 K on Bruker Avance NEO spectrometers, operating at 900 (CAII and ANSII) and 1200 (TTR) MHz, ^1^H Larmor frequency (21.1 and 28.2 T, respectively), equipped with triple resonance cryoprobes. Experiments on encapsulated CAII were also performed on a Bruker Avance NEO spectrometer operating at 600 MHz, ^1^H Larmor frequency (14.1 T), and equipped with a CPQCI cryoprobe.

The NMR samples of the encapsulated proteins were prepared with 540 mm^3^ of RBC suspension (in PBS buffer, pH 7.4) and 60 mm^3^ of ^2^H_2_O, for field frequency lock purposes. The spectra of the free proteins were recorded using the same parameters and buffer composition for comparison purposes. 2D ^1^H–^15^N SOFAST-HMQC experiments^58^ were acquired with an H_N_ offset of 8.1 ppm and an excitation H_N_ bandwidth of 4 ppm, acquisition times of 35 ms (ANSII and CAII) or 20 ms (TTR) and 14 ms (ANSII and CAII) or 10 ms (TTR) for the direct and indirect dimensions, respectively, 256 scans (for the spectra recorded at 900 and 1200 MHz) and 1024 scans (for the spectra recorded at 600 MHz), and interscan delay of 0.2 s. The lower acquisition times used to record the spectra of TTR are related to its shorter transverse relaxation time, due to its high molecular weight and absence of deuteration.

The experiments for the determination of ^15^N transverse relaxation rates were recorded at 310 K and 600 MHz on ^15^N-enriched samples of free CAII in PBS and CAII encapsulated in RBCs. ^15^N transverse relaxation rates (R_2_) were measured using a Carr–Purcell–Meiboom–Gill (CPMG) sequence^59,60^ with delays of 8.48, 16.96, 25.2, 33.92, 42.4, 50.88, 67.84, 84.8, 101.76, 118.72, 144.16, 169.6 ms for the free protein and 8.48, 16.96, 42.4,84.8 and 144.16 ms for the encapsulated protein, with a refocusing delay of 450 μs. Because of the low signal-to-noise ratio, the spectra for the evaluation of ^15^N transverse relaxation rates of the encapsulated protein were acquired using 1024 scans. Transverse relaxation rates were determined by fitting the signal intensities as a function of the delay to a single-exponential decay using Dynamics Center (Bruker) software.

All the spectra were processed on a Bruker TopSpin 4.0 software package and analyzed with CARA program (ETH Zürich).

## Supplementary Material



## Notes

Additional NMR spectra for confirming the success of the washing steps; evaluation of the system stability over time and using different buffers; transverse relaxation analysis for CAII; and comparison of NMR spectra obtained at different magnetic fields (PDF)
