# Supplementary material for: NMR Assessment of the High Order Structure of Biological Therapeutics in Erythrocytes Provides a Tool for Drug Delivery Design
Source: J Am Chem Soc. 2025 Jul 16;147(30):26379–88. doi: 10.1021/jacs.5c05617 (PMC12314899; doi:10.1021/jacs.5c05617)
Supplement: Supplementary file 1 [file ja5c05617_si_001.pdf]

SUPPORTING INFORMATION

NMR Assessment of the High Order Structure of Biological Therapeutics in Erythrocytes Provides a Tool for Drug Delivery Design

Luis Padilla-Cortés ‡[a][b], Giulia Roxana Gheorghita ‡[a][b][c], Francesco Currò[a][b], Rebecca Calamandrei[a][b][d], Bianca Susini[a][b][d], Sara Callozzo[a][b][d], Giulia Crivello[d][e], Pasquale Russomanno[a][b], Enrico Ravera[a][b][d], Linda Cerofolini[a][b][d], and Marco Fragai*[a][b][d]

[a] Department of Chemistry Ugo Schiff (DICUS), University of Florence, Via della Lastruccia 3, 50019, Sesto Fiorentino, Italy.
[b] Centro di Risonanze Magnetiche (CERM), University of Florence, Via Luigi Sacconi 6, 50019, Sesto Fiorentino, Italy.
[c] Giotto Biotech, s.r.l. Via Francesco Crispi 6, 50129Firenze, Italy
[d] Consorzio Interuniversitario di Risonanze Magnetiche di Metalloproteine (CIRMMP), Via Luigi Sacconi 6, 50019, Sesto Fiorentino, Italy.
[e] Department of Agricultural and Food Sciences (DISTAL), University of Bologna, Piazza Goidanich 60, 475121, Cesena, Italy

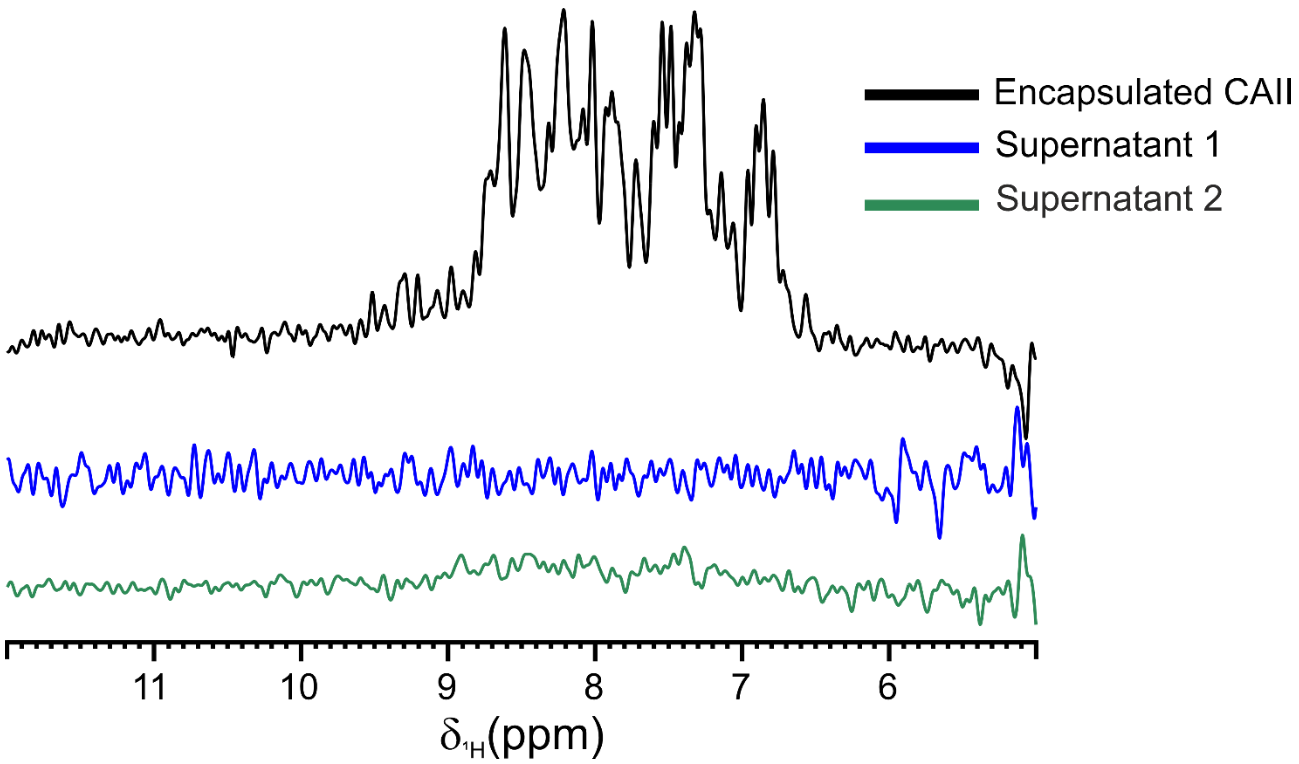

Figure S1. 1st FIDs of the 2D ^{1}H-^{15}N SOFAST-HMQC spectra of a) encapsulated ^{15}N CAII in RBC (black), b) supernatant of the last rinse step (blue, supernatant 1), and c) supernatant recovered from the NMR tube after the acquisition of the set of NMR experiments (lasting 17 h) on CAII encapsulated in RBCs (green, supernatant 2). The data prove that the observed signal in the 2D ^{1}H-^{15}N SOFAST-HMQC spectrum is related to the encapsulated protein only and that the protein is not released from RBCs during the acquisition of the NMR spectra.

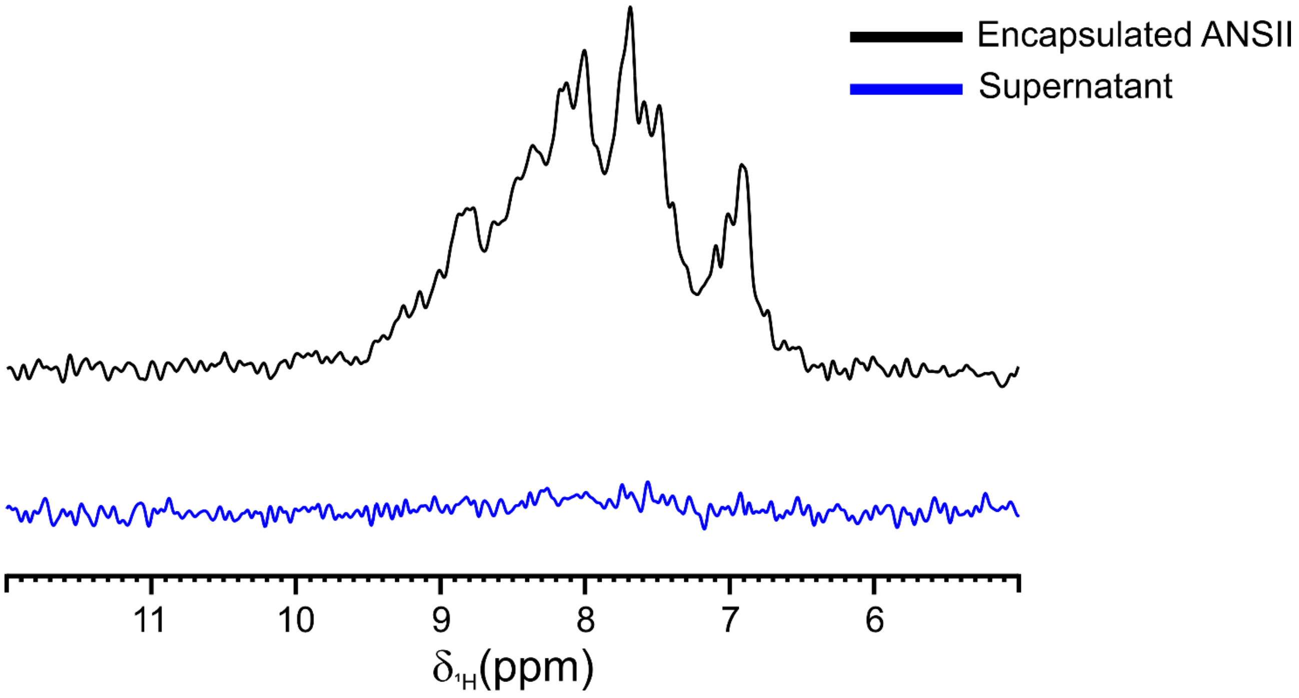

Figure S2. 1st FIDs of the 2D ^{1}H-^{15}N SOFAST-HMQC spectra of encapsulated ^{2}H-^{13}C-^{15}N ANSII in RBCs (black) and supernatant of the last rinse step (blue, supernatant 1).

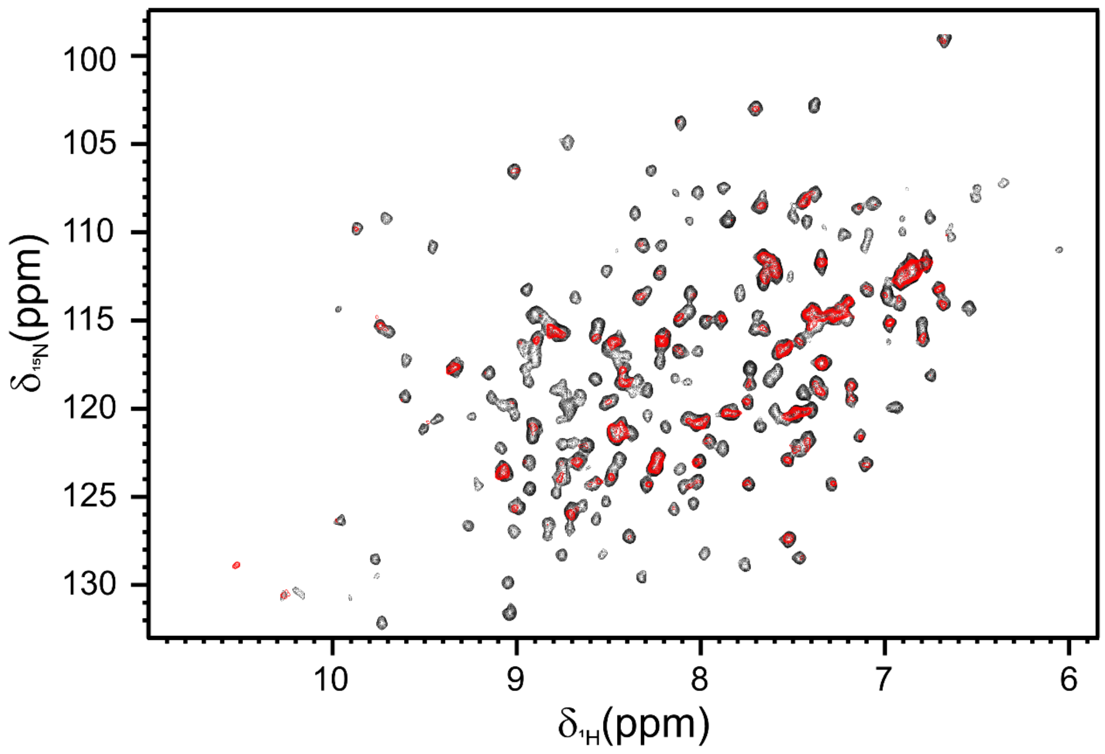

Figure S3. Broadening of NMR signals for CAII encapsulated in RBC over time at 310K. Overlay of 2D ^{1}H-^{15}N SOFAST-HMQC spectra of CAII encapsulated in RBC, acquired immediately after sample preparation (black) and after 2 h of permanence within the magnet at 310 K (red). The spectra were acquired at 310 K on a spectrometer operating at 900 MHz (^{1}H Larmor frequency).

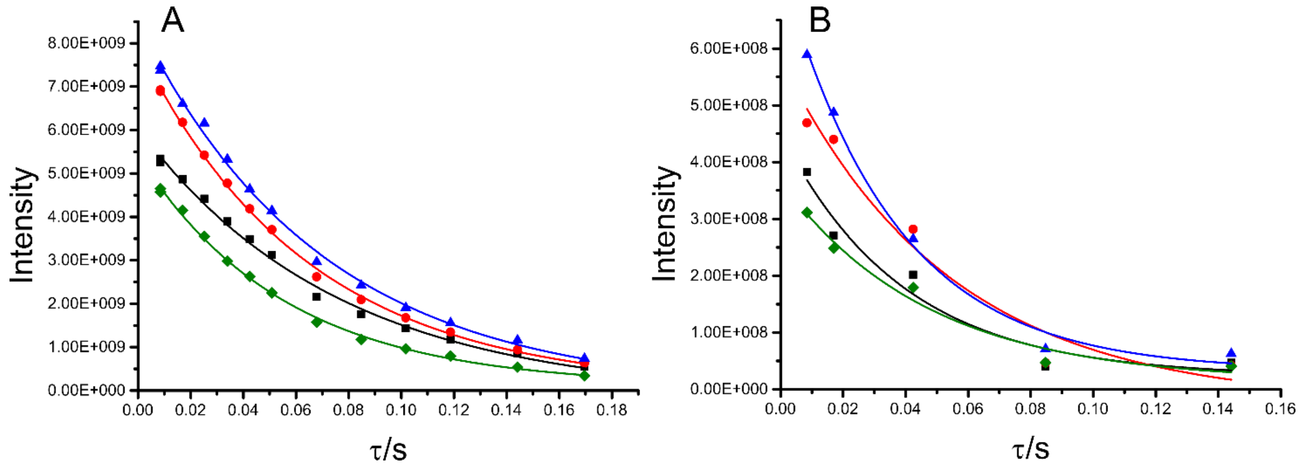

Figure S4. Curves of ^{15}N transverse relaxation. The curves were obtained fitting the signal intensity of the 1st FID of 2D ^{1}H-^{15}N HSQC-type CPMG experiments in Dynamic Center (Bruker) software for (A) free CAII and (B) CAII encapsulated in RBCs.

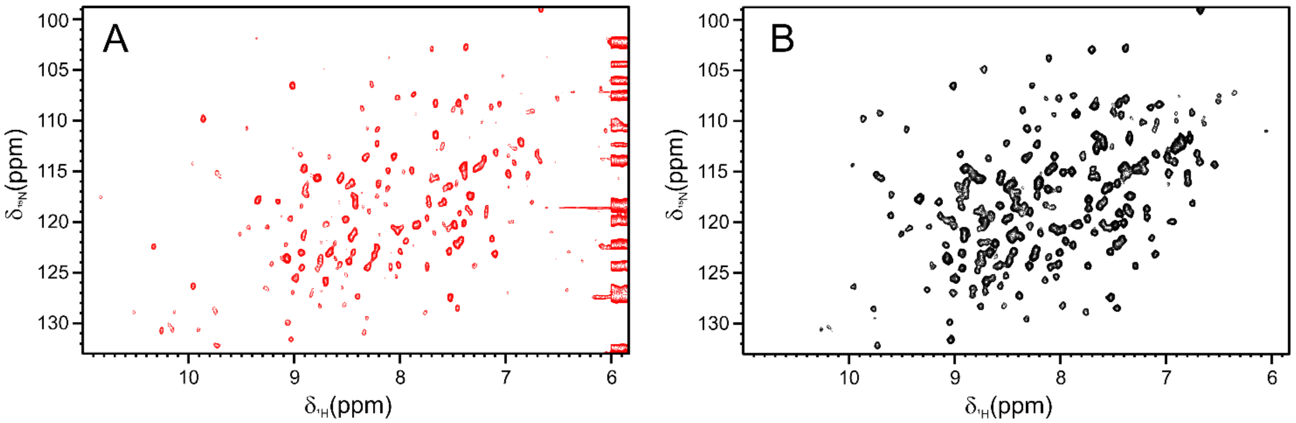

Figure S5. Comparison of 2D ^{1}H-^{15}N SOFAST HMQC spectra recorded on samples of CAII encapsulated in RBCs at (A) 600 MHz (red) and (B) 900 MHz (black). The same acquisition times on the direct and indirect dimensions were used. The spectra were recorded using 1024 and 256 scans for the instrument operating at 600 and 900 MHz, respectively.

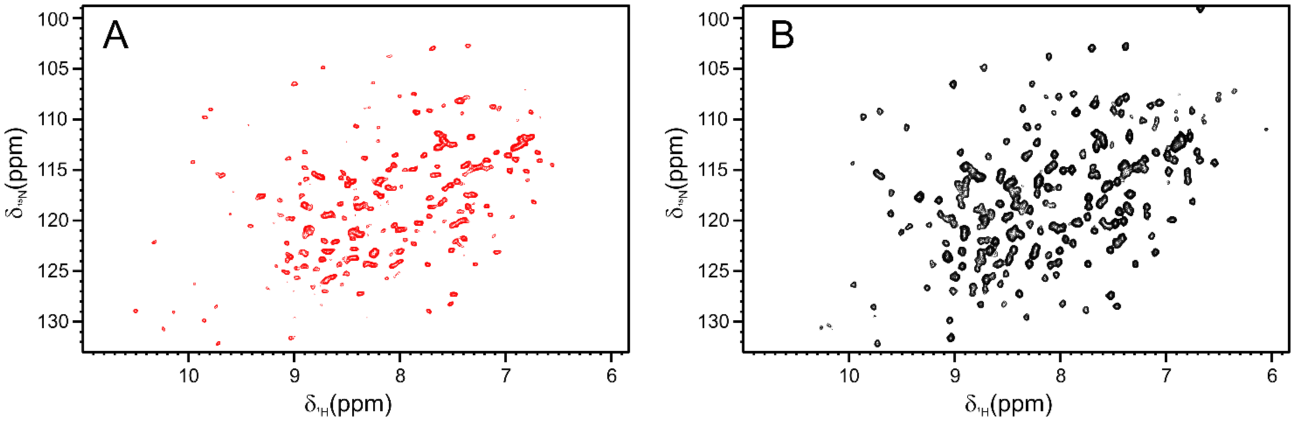

Figure S6. Comparison of 2D ^{1}H-^{15}N SOFAST HMQC spectra recorded on samples of CAII encapsulated in RBCs with (A) or without (B) reduced glutathione (3 mmol·dm^{-3}) and ATP (2 mmol·dm^{-3}) in the buffer. Spectrum (A) was acquired on a spectrometer operating at 950 MHz, while spectrum (B) on a spectrometer operating at 900 MHz. The same acquisition times on the direct and indirect dimensions were used.
